# Recurrent Chyluria Revisited: Lymphangiographic Diagnosis of Pyelolymphatic Fistula With Insights on Endolymphatic Interventions

**DOI:** 10.7759/cureus.86470

**Published:** 2025-06-21

**Authors:** Prudhvinath A Reddy, Sridhar V Prabhu, Yugandhar S, Mithilesh Arumulla, Vikas Kadiyala

**Affiliations:** 1 Department of Radiology, All India Institute of Medical Sciences, Mangalagiri, Mangalagiri, IND; 2 Department of Cardiology, All India Institute of Medical Sciences, Mangalagiri, Mangalagiri, IND

**Keywords:** chyluria, endolymphatic intervention, infective endocarditis, intranodal lymphangiography, lipiodol, lymphangiography, lymphatic imaging, pyelolymphatic fistula

## Abstract

Chyluria, the passage of chyle‐laden lymph into urine, is most frequently linked to filarial infection but may also occur in diverse nonparasitic settings. We describe a 43-year-old man with diabetes, hypertension, and rheumatic heart disease who was admitted with prolonged fever and dyspnea and found to have infective endocarditis. The patient developed persistent milky urine during hospitalization that was unresponsive to a high-protein, low-fat diet supplemented with medium-chain triglycerides. Filariasis serology was negative. Although his comorbidities are not primary etiologic factors for chyluria, the systemic inflammation and lymphatic congestion associated with endocarditis likely triggered central lymphatic obstruction, the reflux of chyle into the renal lymphatics, and chyluria. Diagnostic intranodal lymphangiography with Lipiodol delineated multiple left-sided pyelolymphatic fistulae. No additional embolic agent was administered; nonetheless, Lipiodol's inherent inflammatory and embolic effects led to the complete resolution of chyluria within 24 hours. This case underscores the value of thorough etio-pathological assessment in nonparasitic chyluria, highlights structural lymphatic abnormalities as pivotal contributors to disease persistence, and demonstrates the evolving dual diagnostic-therapeutic role of intranodal lymphangiography. We further review current imaging algorithms and minimally invasive endolymphatic interventions that can obviate surgical treatment in refractory chyluria.

## Introduction

Chyluria is a rare condition characterized by the passage of chyle, rich in triglycerides, chylomicrons, and lymphocytes, into the urine due to an abnormal communication between the lymphatic system and urinary tract, typically at the level of the renal pelvis or ureter [1]. While filariasis remains the predominant cause in endemic regions, nonparasitic etiologies such as trauma, malignancy, thoracic duct obstruction, congenital lymphatic malformations, and systemic infections are increasingly reported [2].

Systemic infections, including septicemia and infective endocarditis, may trigger lymphatic inflammation, obstruction, and reflux into renal lymphatics, resulting in pyelolymphatic fistulae [3]. Diagnosis can be challenging due to the intermittent nature of symptoms and the limited sensitivity of conventional imaging. Recent advances, particularly non-enhanced MRI lymphangiography [4] and intranodal lymphangiography [5], have improved the visualization of the lymphatic system and the detection of occult fistulous tracts.

Importantly, Lipiodol-based intranodal lymphangiography not only provides anatomical detail but also can promote spontaneous resolution through the inflammatory embolization of the leaking channels [6]. Emerging interventional strategies such as thoracic duct stenting [7] and renal-lymphatic embolization [8], along with advanced diagnostic techniques such as contrast-enhanced lymphosonography [9], are expanding the management options in complex or refractory cases.

This case is noteworthy due to the rare occurrence of multiple pyelolymphatic fistulae secondary to a systemic infection and the complete resolution of symptoms following diagnostic lymphangiography alone, without the need for further invasive intervention. It underscores the diagnostic and therapeutic utility of Lipiodol-based lymphangiography and highlights its role in the evolving management of nonparasitic chyluria.

## Case presentation

A 43-year-old man with known diabetes, hypertension, and rheumatic heart disease presented with a three-month history of intermittent high-grade fever with chills and rigors, along with progressive dyspnea on exertion. Clinical examination and echocardiography revealed a mass lesion on the anterior mitral leaflet, leading to a diagnosis of infective endocarditis. Blood cultures grew *Streptococcus gordonii*, and he was started on intravenous antibiotics.

The patient developed milky white urine approximately one week into hospitalization. Urine examination revealed elevated triglyceride levels (150 mg/dL), confirming the diagnosis of chyluria. Conservative dietary measures consisting of a high-protein, low-fat diet supplemented with medium-chain triglycerides were initiated and continued for seven days, but symptoms persisted. Filariasis serology yielded negative results. Given the patient's systemic inflammatory state due to infective endocarditis, lymphatic inflammation and obstruction were considered plausible contributors. Due to persistent chyluria, a decision was made to proceed with intranodal lymphangiography. This technique was selected over traditional pedal lymphangiography due to its minimally invasive nature, better patient comfort, and the ability to rapidly visualize central lymphatics and the better delineation of lymphatic anatomy. Computed tomography (CT) urography was deferred due to its limited sensitivity for detecting small-volume pyelolymphatic fistulae.

**Figure 1:**
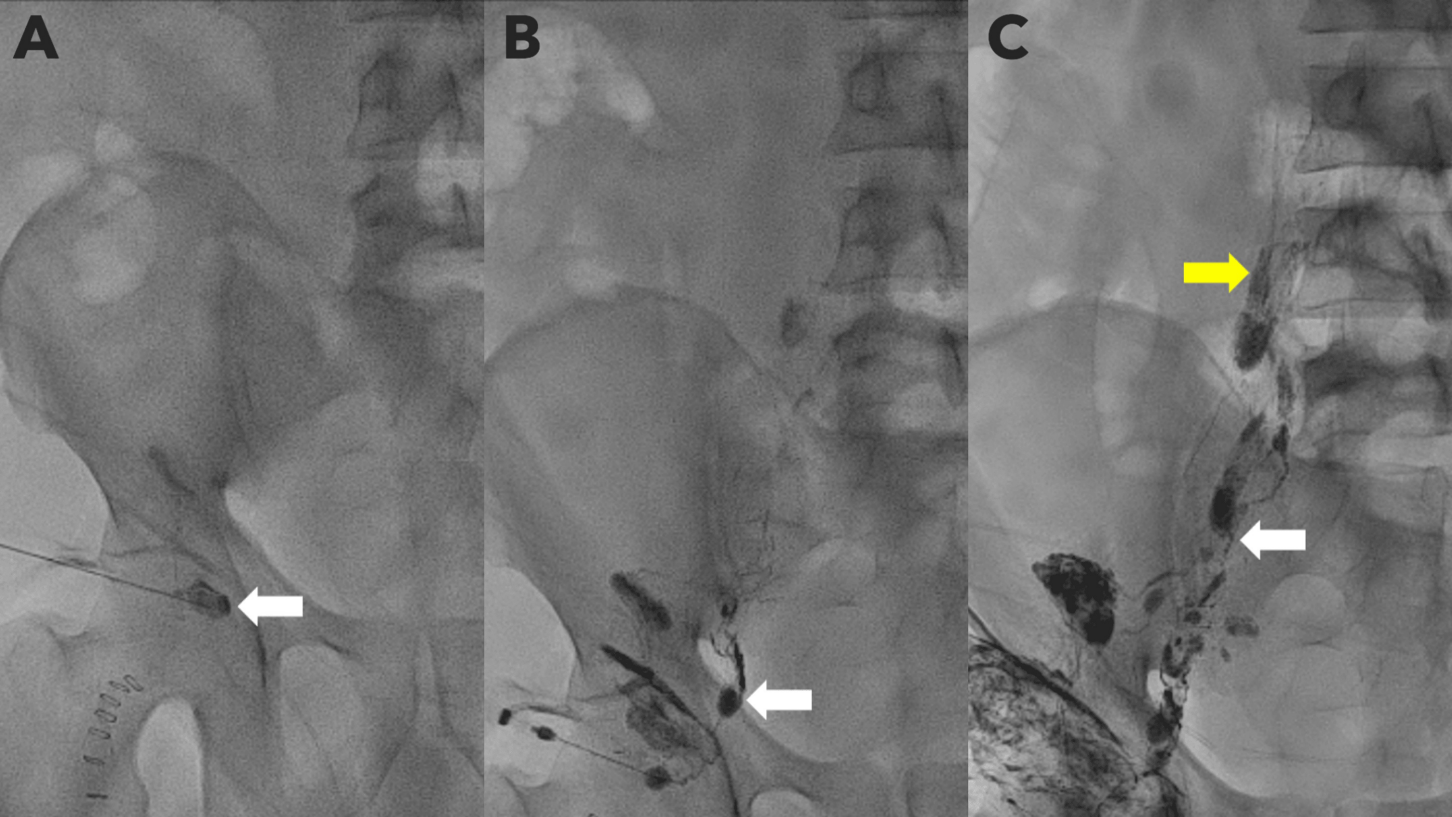
Spot fluoroscopic images during lymphangiography demonstrating the antegrade progression of Lipiodol through the lymphatic system. (A) Lipiodol injection into the right inguinal lymph node (white arrow). (B) Opacification of the right inguinal lymphatic channels (white arrow). (C) Continued progression with opacification of the common iliac (white arrow) and para-aortic lymph node chains (yellow arrow).

Under ultrasound guidance, the most prominent right inguinal lymph node was punctured using a 22 G lumbar puncture (LP) needle. Lipiodol was then slowly injected under fluoroscopic guidance (Figure 1A). The antegrade progression of Lipiodol through the right inguinal lymphatics was continuously monitored (Figure 1B). After 90 minutes, there was opacification of the central abdominal lymphatic channels (Figure 1C), with reflux into the left renal lymphatics observed by three hours.

**Figure 2:**
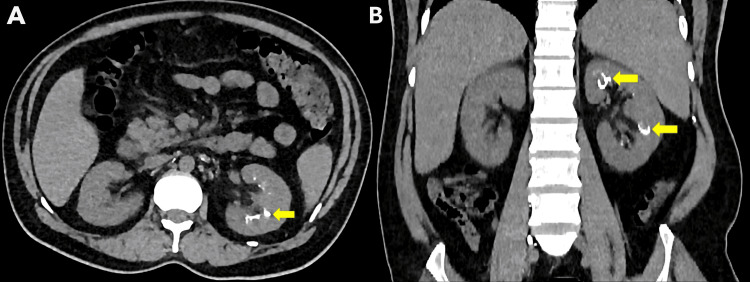
Axial (A) and coronal (B) computed tomography (CT) images obtained 12 hours after lymphangiography, demonstrating Lipiodol opacification within multiple sites of the left pelvicalyceal system (yellow arrow), confirming the presence of multiple pyelolymphatic fistulae.

Follow-up computed tomography (CT) scan obtained 12 hours after the procedure demonstrated Lipiodol opacification at multiple sites within the left pelvicalyceal system, confirming the presence of multiple pyelolymphatic fistulae (Figure 2).

The patient's chyluria resolved completely within 12 hours of the lymphangiography, and follow-up over the next 72 hours showed the normalization of urine color and consistency. Urinary triglyceride levels dropped from 150 mg/dL to <20 mg/dL. No recurrence was observed at the three-month clinical review. 

## Discussion

Chyluria is characterized by the presence of chyle, a lymphatic fluid rich in albumin, emulsified fats, and fibrin, in the urine, resulting from abnormal lymphatic-urinary communication, typically occurring at the level of the renal pelvis [1]. While parasitic infections, especially filariasis, remain the most common cause worldwide, systemic infections have also been implicated as important nonparasitic etiologies of chyluria [2]. In such cases, inflammation and lymphatic obstruction secondary to infection can increase lymphatic pressure, leading to retrograde flow and the formation of lympho-urinary fistulae. In our patient, septicemia secondary to infective endocarditis, a complication of preexisting rheumatic heart disease, is believed to be the underlying cause of chyluria, likely due to lymphatic obstruction and inflammation leading to the reflux of chyle from the central lymphatics to the renal lymphatics and pyelolymphatic fistulae formation. Importantly, while no formal embolization was performed, lymphangiography alone led to the complete resolution of symptoms, likely through Lipiodol-induced inflammatory occlusion. This case exemplifies the evolving therapeutic utility of lymphangiography in managing lymphatic leaks.

Diagnostic imaging plays a vital role in detecting and localizing lymphatic leaks. Intranodal lymphangiography, particularly when combined with post-lymphangiographic computed tomography (CT), remains the preferred method for delineating the size, number, and site of lymphatic fistulae [5].

In addition to conventional intranodal lymphangiography, noninvasive imaging techniques such as MRI lymphangiography have emerged as useful tools for delineating central lymphatic anatomy. MRI lymphangiography, particularly with heavily T2-weighted or contrast-enhanced sequences, provides valuable anatomical detail without the risks associated with iodinated contrast, making it suitable for select patients with contraindications to fluoroscopic procedures or when thoracic duct anomalies are suspected [10]. However, its sensitivity in detecting active lymphatic leaks remains limited compared to fluoroscopic techniques [4].

Beyond diagnosis, lymphangiography has demonstrated therapeutic benefits. In the present case, chyluria was temporally associated with the onset of systemic infection, and we suspect that the inflammatory milieu of infective endocarditis led to secondary lymphatic obstruction and pyelolymphatic fistula formation. While intranodal lymphangiography was initially pursued for diagnostic purposes, the resolution of chyluria suggests a therapeutic effect from Lipiodol. Prior studies have shown that Lipiodol can cause localized inflammatory reactions and embolization of leaking lymphatic channels, a mechanism likely operative here given the rapid symptom resolution without further intervention [6]. The spontaneous resolution of chylous leaks following lymphangiography alone has been documented in prior literature, further supporting its potential therapeutic effect [11].

Management strategies vary depending on severity. Mild to moderate chyluria may be addressed conservatively through dietary modifications and monitoring [1]. However, severe or refractory cases require more invasive interventions. We clarify that while only diagnostic intranodal lymphangiography was performed in this patient, the interstitial and retrograde lymphatic embolization techniques described herein are presented for educational purposes. These options are particularly relevant for patients with persistent or recurrent symptoms after diagnostic imaging. Advances in lymphatic imaging have revived interest in minimally invasive, image-guided techniques that directly target lymphatic leaks.

Thoracic duct stenting has also been reported in the literature as an emerging option in cases with central lymphatic obstruction, particularly at the thoracic duct-venous junction. By relieving obstruction and restoring antegrade lymphatic flow, stenting can reduce lymphatic hypertension and promote distal drainage [7].

Interstitial lymphatic embolization involves the percutaneous injection of embolic agents, such as N-butyl cyanoacrylate (NBCA) mixed with Lipiodol, into lymph nodes or perilymphatic tissues supplying the fistula. Nguyen et al. successfully utilized this technique under the balloon occlusion of the thoracic duct to prevent inadvertent glue reflux and preserve ductal patency, achieving symptom resolution in treated cases [5].

Retrograde thoracic duct access enables catheterization via either direct percutaneous puncture (typically under ultrasound guidance) or intravenous retrograde cannulation through the left subclavian vein. This approach is particularly useful when conventional lymphangiography fails to delineate clear targets. Hur et al. reported complete or significant symptom improvement after superselective embolization of aberrant renal lymphatics using NBCA-Lipiodol mixtures via this approach [12].

Both interstitial and retrograde embolization techniques offer precise, minimally invasive treatment with reduced morbidity compared to surgical options. Despite its utility, lymphangiography is not without limitations. Challenges include failure to visualize slow-flow or intermittent leaks, potential allergic reactions to iodinated contrast, and rare complications such as extravasation, fat embolism, or lymphangitis. Similarly, lymphatic embolization techniques, though promising, carry risks of glue reflux, ductal injury, or nontarget embolization.

Given the possibility of recurrence, patients require clinical follow-up. Imaging may be considered in symptomatic cases to evaluate for fistula persistence or reformation. The current patient remains asymptomatic at three months, but long-term surveillance is warranted in these cases.

Selection should be tailored based on anatomical findings, leak accessibility, and clinical context. While this patient was managed by an interventional radiology and cardiology-led team, we recommend a multidisciplinary approach in recurrent or complex chyluria, involving interventional radiology, urology, nephrology, and infectious disease specialists to optimize outcomes and guide tailored management.

## Conclusions

This case highlights a rare instance of nonparasitic chyluria secondary to infective endocarditis, with complete resolution following diagnostic intranodal lymphangiography alone. The findings underscore the diagnostic and occasional therapeutic potential of Lipiodol-based lymphangiography in managing lymphatic-urinary fistulae. Minimally invasive imaging-guided techniques offer effective alternatives to surgery in select patients with persistent or recurrent chyluria. Long-term follow-up is essential to monitor for recurrence, and a multidisciplinary team is recommended for complex cases to ensure accurate diagnosis, comprehensive management, and improved clinical outcomes. Continued advances in lymphatic imaging may further expand treatment options for complex cases.
